# Regenerative Strategies in Dentistry: Harnessing Stem Cells, Biomaterials and Bioactive Materials for Tissue Repair

**DOI:** 10.3390/biom15040546

**Published:** 2025-04-08

**Authors:** Vidhya Rekha Umapathy, Prabhu Manickam Natarajan, Bhuminathan Swamikannu

**Affiliations:** 1 Department of Public Health Dentistry, Thai Moogambigai Dental College and Hospital, Dr. M.G.R. Educational and Research Institute, Chennai 600107, Tamil Nadu, India; 2 Department of Clinical Sciences, Centre of Medical and Bio-Allied Health Sciences and Research, Ajman University, Ajman P.O. Box 346, United Arab Emirates; 3 Department of Prosthodontics, Sree Balaji Dental College and Hospital, Bharath Institute of Higher Education and Research, Pallikaranai, Chennai 600100, Tamil Nadu, India; bhumi.sbdch@gmail.com

**Keywords:** regenerative dentistry, stem cells, biomaterials, tissue regeneration, dental pulp, periodontal ligament

## Abstract

Advanced bioengineering, popularly known as regenerative dentistry, has emerged and is steadily developing with the aim of replacement of lost or injured tissues in the mouth using stem cells and other biomaterials. Conventional therapies for reparative dentistry, for instance fillings or crowns, mainly entail the replenishment of affected tissues without much concern given to the regeneration of tissues. However, these methods do not enable the natural function and aesthetics of the teeth to be maintained in the long term. There are several regenerative strategies that offer the potential to address these limitations to the extent of biologically restoring the function of teeth and their components, like pulp, dentin, bone, and periodontal tissues. Hence, stem cells, especially dental tissue derived stem cells, such as dental pulp stem cells, periodontal ligament stem cells, or apical papilla stem cells, are quite promising in this regard. These stem cells have the potentiality of generating precise dental cell lineages and thus are vital for tissue healing and renewal. Further, hydrogels, growth factors, and synthetic scaffolds help in supporting the stem cells for growth, proliferation, and differentiation into functional tissues. This review aims at describing the process of stem cell-based tissue repair biomaterials in dental regeneration, and also looks into the practice and prospects of regenerative dentistry, analysing several case reports and clinical investigations that demonstrate the efficacy and limitations of the technique. Nonetheless, the tremendous potential for regenerative dentistry is a reality that is currently challenged by biological and technical constraints, such as scarcity of stem cell sources, inadequate vascularization, and the integration of the materials used in the procedure. As we move forward, the prospects for regenerative dentistry are in subsequent developments of stem cell technology, biomaterial optimization, and individualized treatment methods, which might become increasingly integrated in dental practices globally. However, there are regulatory, ethical and economic issues that may pose a hurdle in the further advancement of this discipline.

## 1. Introduction

Regenerative strategies in dentistry are revolutionizing traditional dental care by focusing on repairing, restoring, and regenerating damaged or lost dental tissues, including enamel, dentin, pulp, and periodontal structures [1]. Traditionally, dentistry has been concerned with filling procedures, crowning and bridgework, and root canal treatment. As in the case of lambs, these methods of re-establishing function failed to restore tissues at the same level at which they replaced structures. For example, the very common dental filling material that can treat a cavity, which is either an amalgam or a composite resin, or even gold, cannot rebuild the missing tooth structure and can pose problems, such as wear, crack or recurrent decay [2]. Likewise, crowns and bridges restore areas of the mouth that are missing or have injured teeth; however, these restorations demand extensive removal of healthy tooth tissue and have limited resistance to damage. Despite the usefulness of endodontic therapy in managing a diseased tooth and retaining it within a dental arch, this makes the tooth non-vital and brittle, due to extraction or degeneration of the pulp tissue that brings nutrients and sensitivity [3]. Furthermore, some of these treatments may comprise biological incompatibility, which may cause side effects such as allergic reactions, hence the need for more biologically compliant solutions.

Due to the globally ageing population and dental diseases including caries and periodontal diseases, the need for an efficient and more environmentally friendly approach is increasing [4]. Thus, the attractive concept of regenerative dentistry is being developed as an answer to these challenges, mainly based on biologically created tissue repair mechanisms. Stem cell therapy, tissue engineering and bioactive materials are used in an attempt to recreate the normal mechanical and structural properties of the dental hard tissues, thereby accounting for the shortcomings of conventional restorative procedures [5]. For instance, stem cell applications have indicated the capability of reconstructing dental pulp, whereas bioactive materials have been identified as having re-healing properties in reconstructing both tooth dentin and enamel [6]. Techniques of periodontal regeneration are also constantly improving, which provides treatment solutions for regeneration of the supporting tissues of the teeth, i.e., gums and alveolar bone, which are usually affected by periodontal diseases.

The transition to regenerative dentistry is, therefore, not only a reaction to the ineffectiveness of conventional practices but, rather, a chance to disrupt the dental field [7]. These strategies directly address biologically incorporated forms of treatment that can minimize the invasiveness of processes, the results of which are long-lasting, and elevate the overall level of patient care. Obviously, they can also contribute to reducing the harm that dental materials cause to the environment and reducing the frequency of procedures for patients’ safety and environmental conservation [8].

Enamel, dentin, dental pulp, and periodontal tissues quickly go missing following destruction, because they have a restricted capacity for self-repair [9]. This raises concern, especially in managing diseases such as caries, pulpitis, and periodontitis, as well as traumatic injuries. In order to address these problems, regenerative dentistry aims at achieving its goals based on achievements in the field of stem cell biology, bioengineering scaffolds, and growth factors, in order to reconstruct damaged tissue organization and function [10]. Dental stem cells include dental pulp stem cells (DPSCs), periodontal ligament stem cells (PDLSCs), and stem cells from exfoliated deciduous teeth (SHED), which are leading in this realm of innovation. These cells are capable of differentiating into a variety of dental-specific cell phenotypes, including odontoblast-like cells or periodontal fibroblasts, thus offering the potential for new tissue engineering applications [11]. These therapies put into use bioactive materials, such as hydrogels, nanocomposites and scaffolds, which, when used in combination with these therapies, foster tissue regeneration in a natural manner.

This emerging paradigm also correlates with a number of other trends that reflect a certain disillusionment with the traditional framing of dentistry. Reconstructive strategies are frequently based on synthetic materials that are incapable of mimicking the dynamic structure and self-healing capacity of healthy tissues [12]. Moreover, traditional approaches applied often entail the destruction of healthy tissue, leading to reduced stability and a more limited prognosis in the long run. Modern techniques used in natural regeneration, therefore, provide a viable solution that is ecological and concordant with biological functionality [13].

The goals of regenerative dentistry are ambitious yet transformative. Unlike classical reparative therapies that aim at mimicking the lost or damaged tissues, regenerative strategies are aimed at recreating the morphogenetic profile of dental tissues and their reciprocal interactions within the oral context [14]. Stem cell-based approaches, combined with growth factors and biomaterial applications, allow the addressing of enamel and dentin regeneration, as well as of pulp and periodontal structures. All these strategies follow the principles of tissue engineering by achieving integration with surrounding tissues alongside functional characteristics, such as sensation, vascular supply, and the immune system, thus giving patients better oral health, as well as quality of life [15]. Thus, regenerative dentistry holds promise as a more progressive paradigm in advanced dental practice, which is aimed at solving many of the issues that stem from using the conventional non-neural repair approach while enhancing tissue regeneration. This approach draws on the principles of stem cell biology, tissue engineering, and biomaterials science to revolutionize clinical dentistry, at the same time enhancing the prognosis for patient care.

## 2. Stem Cells in Dental Tissue Regeneration

Stem cells are undifferentiated cells having the ability to differentiate into many cell types within a particular cell lineage. Among all cells, stem cells are the most promising in the context of regenerative dentistry because these cells can engage both in self-renewal and differentiation to produce cells required for the effective repair of tissues [16]. Moreover, these cells have the capability of regenerating dental structures, like pulp, dentin, periodontal tissues, and alveolar bones. Sources of stem cells employed in dental regeneration can be classified into dentogenesis-derived stem cells and non dentogenesis-derived stem cells, which makes them quite diverse and suitable for use in a distinct manner to repair afflicted oral tissues [17].

These stem cells are sourced from human dental pulp stem cells (DPSCs), which can help in formation of dentin and pulp tissue whereas stem cells from apical papilla (SCAPs) help in the formation of roots and repair of pulp [18]. Several types of stem cells have been found; periodontal ligament stem cells (PDLSCs) which self-renew and differentiate into periodontal ligament, and alveolar bone and dental follicle progenitor cells (DFPCs), which facilitate periodontal and bone formation (Table 1). Research has shown that stem cells from human exfoliated deciduous teeth (SHED) offer a robust prospect in dentin and connective tissue engineering [19]. These stem cells, when teamed with biomaterials and signalling molecules, proffer tissue specific and efficacious solutions to dental tissue regeneration.

### 2.1. Types of Stem Cells Utilized in Dental Tissue Regeneration

#### 2.1.1. Dental-Derived Stem Cells

Dental derived stem cells are sourced directly from dental tissues and are known for their tremendous capability in tissue engineering [20]. Dental pulp stem cells (DPSCs) derived from the dental pulp of permanent teeth are considered suitable for pulp and dentin regeneration due to their high proliferative potential and ability to differentiate into odontoblast-like cells. SHED cells have been derived from harvested baby teeth and have proved to be very functional in tissue regeneration, and specifically in the formation of dentin and connective tissues [21]. PDLSCs are more involved in the repair process or formation of new periodontal tissue, such as ligament, cementum, and alveolar bone, which makes these cells therapeutic for periodontal diseases [22]. Likewise, the apical papilla stem cells (SCAPs) present in the root tip of the teeth of the developing stage immature teeth are involved in root development and cell differentiation, evidencing future potential in pulp and dentin regeneration [23]. It has been revealed that dental follicle progenitor cells (DFPCs) derived from the dental follicle of developing teeth are capable of differentiating into osteoblasts and fibroblasts involved in periodontal and bone formation [24].

#### 2.1.2. Non-Dental Mesenchymal Stem Cells (MSCs)

Other mature human somatic stem cells are sourced from different regions within the body and provide a balance to the dental-derived stem cells when used in tissue engineering. MSCs obtained from bone marrow are noted for their ability to differentiate into osteogenic cells and are thus useful in augmenting alveolar bone repair [25]. MSCs derived from adipose tissue, ADSCs, have the proven potential of differentiation into dental-related cell lineages under certain conditions for periodontal and pulp tissue engineering. MSCs derived from the umbilical cord also have potential use in periodontal/bone tissue engineering and have advantages over bone marrow-derived MSCs, as the procedure is less invasive.

#### 2.1.3. iPSCs (Induced Pluripotent Stem Cells)

**Figure 1:**
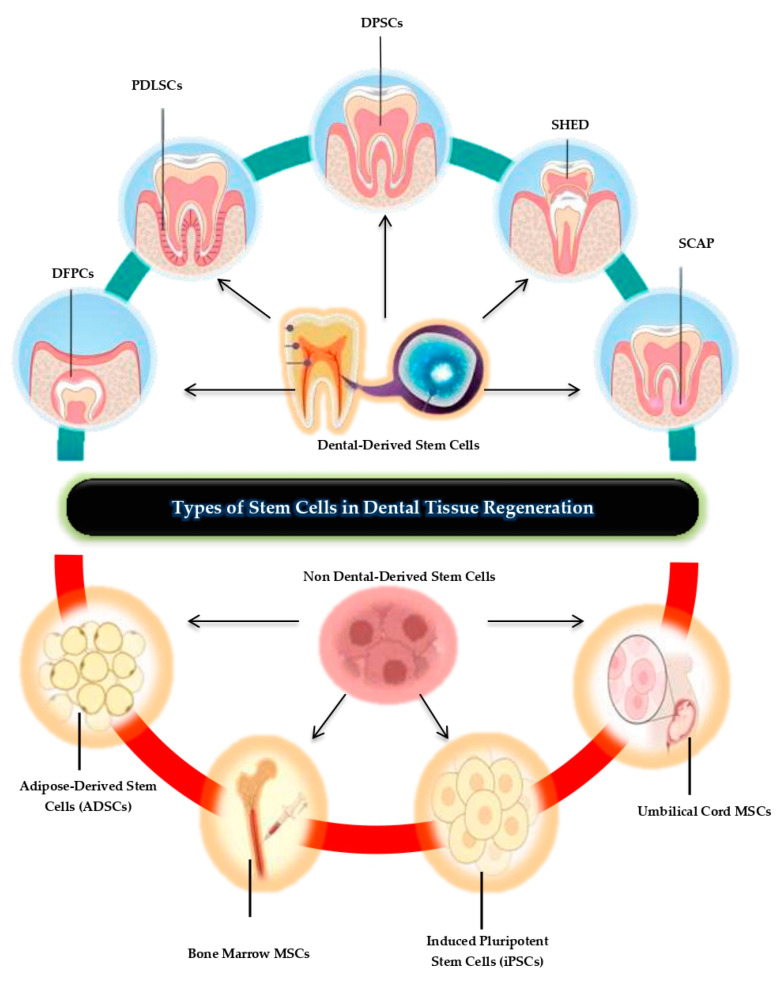
Types of stem cell in dental tissue regeneration: Dental pulp stem cells (DPSCs) regenerate pulp and dentin due to their high proliferation and odontoblast differentiation. SHED (from baby teeth) supports dentin and connective tissue repair. PDLSCs regenerate periodontal tissues, like ligaments, cementum, and bone. SCAP aids in root development and pulp/dentin repair, while DFPCs contribute to bone and periodontal tissue formation. Non-dental MSCs, such as bone marrow MSCs, repair alveolar bone, while adipose- and umbilical-derived MSCs enhance periodontal and pulp tissue engineering.

This is a process in which human adult differentiated cells are forced to revert back to stem cells with the ability to differentiate into any desired cell type, including odontogenic cells (Figure 1). In dental tissue engineering, iPSCs are considered valuable due to their pluripotency and the ability to create individualized treatments [26]. Thus, based on the properties of these various stem cell types, dental regenerative processes can address a large variety of diseases and injuries that can affect the oral cavity, providing for patient-specific efficient treatment protocols for the regeneration of tissues in the oral cavity.

### 2.2. Mechanisms Underlying Stem Cell-Mediated Tissue Repair and Regeneration

**Figure 2:**
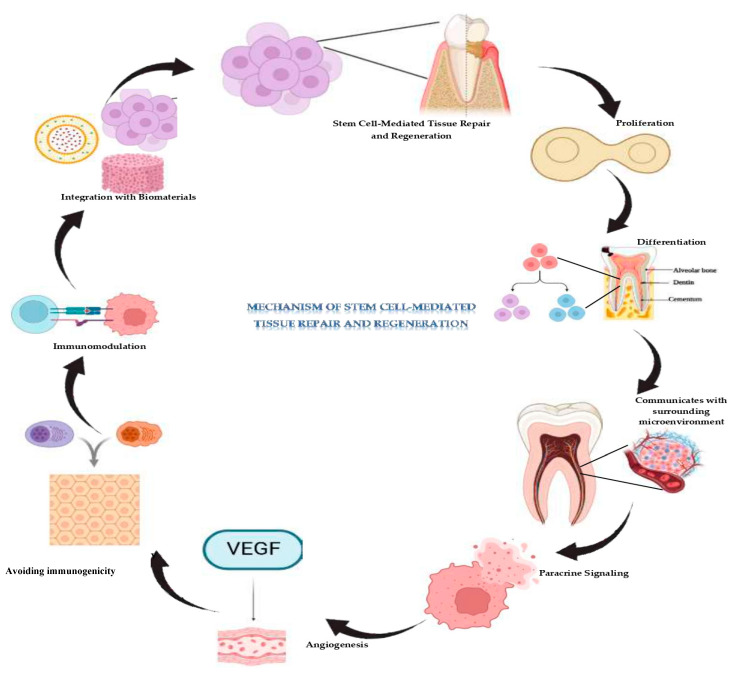
Mechanism of stem cell mediated dental tissue regeneration. Stem cell-mediated dental tissue repair involves stem cells proliferating to replace damaged cells, and differentiating into specific cells, like odontoblasts, cementoblasts, fibroblasts, and osteoblasts, for tissue regeneration. They interact with the microenvironment, releasing growth factors and VEGF to promote cell growth, angiogenesis, and resident cell activation. Stem cells also reduce inflammation and recruit immune cells for healing. However, challenges, such as safety, biomaterial integration, and scalability need to be addressed for clinical success.

Stem cell characteristics for tissue repair and regeneration strongly depend on their quality to proliferative, differentiate and dynamically communicate with their surrounding environment (Figure 2). Activated by injury or damage, stem cells increase their numbers and move towards the injured area with a direct supply of a constant stream of cellular components that are necessary for tissue regeneration [27]. These cells turn into dental-specific cell lineages, like odontoblasts, which contributes to the formation of dentin, cementoblasts, which contribute to cementum, fibroblasts, which form connective tissue, and osteoblasts, which form alveolar bones [28].

Besides the featured differentiation, stem cells perform paracrine signalling, releasing bioactive molecules that upgrade their regenerative ability. These include growth factors, cytokines and extracellular vesicles that stimulate and regulate the cellular activities in the surrounding stroma [29]. By such signalling, stem cells induce new blood vessel formation, activate resident cells and attract immune cells for tissue repair. Angiogenic factors, including VEGF produced by stem cells, aim at the formation of new blood vessels needed to supply the regenerating tissue with requirements for sustenance, oxygen and nutrients to support growth and repair [30].

Stem cells also have immunomodulatory potential, which minimizes inflammation and prepares the area for tissue reconstruction. They cater for the appropriate regulation of immune response, reduce in essence chronic inflammation, and optimally stimulate the healing process for maximum regenerative efficiency [31]. Collectively, these mechanisms allow stem cells not only to replace lost tissues and tissues that have been damaged, in the teeth but also reinstate them to functionality. Most importantly, their proven efficacy in modulating cellular activities for tissue formation, stimulating blood supply, and controlling inflammation makes them central to future developments in regenerative dentistry.

Stem cell-based therapies offer transformative potential for dental regeneration but face challenges., like ensuring safety, particularly avoiding tumorigenicity, and controlling cell differentiation. Scalability, standardization, and ethical considerations also need resolution, especially in sourcing and expanding stem cells reliably [32]. Integration with biomaterials to create supportive scaffolds for tissue regeneration remains complex but vital. Despite these hurdles, stem cell therapies promise personalized treatments and the ability to regenerate complex dental tissues, offering sustainable and biologically integrated solutions. Ongoing research is key to addressing these barriers and realizing their clinical potential.

## 3. Biomaterials in Dental Regeneration

Biomaterials are fundamental for dental regeneration because they offer the proper structural framework needed to support cellular mechanisms involved in tissue repair [33]. When used in conjunction with stem cells and growth factors, these materials also provide the necessary scaffolding for the growth of new dental tissues, like dentin, pulp, periodontal ligament, and alveolar bone. Recent advancements in biomaterial sciences have increased the possibility of restoring both the form and function of compromised dental tissues, going beyond the shortcomings of conventional restorative approaches.

### 3.1. Types of Biomaterials Used in Dental Regeneration

Stem cell therapies and tissue repair in dental restoration require biomaterials that aid in regeneration. Hydrogels are water-swollen, three-dimensional cross-linked hydrophilic polymer networks that mimic the ECM, and their applications are in the regeneration or repair of soft tissues, like dental pulp and periodontal ligament [34]. They can easily support cell adhesion, proliferation and differentiation and can be mixed with stem cells or growth factors to aid in regeneration.

Chemotactic factors, based on the soluble proteins that influence cell functions, including migration and differentiation, are the dominant component in growth. In dentistry, BMPs, PDGF, VEGF and TGF-β are some of the growth factors used in dental applications to regenerate bones, periodontium and dental pulp [35]. Scaffold materials that are artificial or synthetic can be designed or fabricated to suit the required dental application.

Other examples of biodegradable polymeric scaffolds include PLA, PGA, and PLGA, which have been used in bone and periodontal regeneration. The incorporation of synthetic polymers with inorganic material improves the mechanical properties of the scaffold and bioactivity of HA [36]. Bioactive ceramics and electro-spun nanofibers also provide frameworks close to the native bone tissue and soft tissues, which can support cell migration, cell attachment, and differentiation [37]. Hydrogels, growth factors and synthetic scaffolds improve the applications of dental regenerative medicine by merging them with stem cell-based therapies in order to improve clinical applicability and the outcomes of dental regenerative medicine.

### 3.2. Advances in Biomaterial Design for Enhanced Dental Tissue Repair

New developments in biomaterial design for dental tissue repair have greatly improved regenerative medicine. Other advanced biomaterials include thermo-sensitive hydrogels, which can load the bioactive molecules and release them in response to a stimulus and thereby enhance dental pulp regeneration [38]. Nanomaterials, such as nanohydroxyapatite and nano-silver, show improved mechanical properties and antimicrobial properties, which promote bone formation and prevent infection.

3D printed biomaterials enable the development of customized, individualized scaffolds that involve stem cells for tissue engineering. Bioactive glasses and ceramics are biomimetic materials that mimic the structure of tissues involved in the bonding of bones through osteogenesis [39].

Composite materials, containing synthetic and natural components, may have adjustable characteristics for bone, dentin, and periodontal tissue engineering (Table 2). Intrinsic characteristics, such as biocompatibility, biodegradability, and mechanical properties, will help these materials to determine the success of the integration and regeneration of tissues. These are initiating changes in dental regenerative therapies, offering better, long-lasting, and patient-specific treatment methods for damaged dental tissues.

## 4. Techniques in Regenerative Dentistry

### 4.1. Protocols for Incorporating Stem Cells and Biomaterials in Dental Repair

The conceptual frameworks and significance of stem cells and biomaterials are noteworthy and provide guidelines for dental tissue regeneration. Dental stem cells can be harvested from tissues, such as the pulp or periodontal ligament, then extracted and classified based on differentiation potential [40]. Hydrogels, synthetic scaffolds, and bioactive ceramics make up the bioactive materials that are used depending on the tissue to be regenerated, since they provide mechanical support to the cells, allow attachment of stem cells, and release molecules that support tissue healing. The cells are cultured on scaffolds, implanted in the injury site and then observed for tissue formation and apposition [41]. These growth factors are then introduced to act as aids to differentiation and maturation processes.

### 4.2. Application of Platelet-Rich Plasma (PRP) and Platelet-Derived Growth Factors

PRP stands for platelet rich plasma, a concentrated autologous plasma with platelet counts 1.5–2 times higher than baseline platelet concentration, containing growth factors, such as PDGF, TGF-β and VEGF, responsible for stimulating cell proliferation, migration and differentiation. PRP is prepared from the patient’s blood and then can act directly on the sites of lesions or in combination with bioactive materials to stimulate tissue repair [41]. It enhances angiogenesis and supports cell differentiation and chemo-taxis, helps in regeneration of the periodontal ligament and bone, and is of great importance in osseo-integration of dental implants.

The process of integration of stem cells, biomaterials and growth factors improves the options for stratified component regeneration. For instance, the combination of stem cells with biomaterials containing growth factors, like BMPs or PDGF, enhances tissue regeneration. This mutualistic approach helps in proper differentiation of stem cells and biomaterials give the necessary structural framework to support the formation of the tissues [42]. This is because PRP acts to enhance healing, angiogenesis, and stem cell differentiation at a much faster rate. Newer techniques, such as gene therapy and bioprinting, are on the horizon for dTTR, which bring even greater personalization and more accurate approaches to treatment. These approaches, when applied together, lay the foundation for regenerative dentistry to deliver even more targeted and effective solutions for oral care problems in the future.

### 4.3. Tissue Engineering and Biomolecules in Dentistry

Tissue engineering in dentistry is fundamentally built upon the triad of scaffolds, cells, and signalling molecules, which collectively drive effective tissue repair and regeneration. While the initial focus of this review emphasized stem cells and biomaterials, bioactive molecules and growth factors play a pivotal role in directing cellular behaviour, differentiation, and extracellular matrix deposition. Key bioactive molecules involved in this tissue engineering include BMPs, TGF-β, FGF, VEGF, PDGF [43].

Bone Morphogenetic Proteins (BMPs), particularly BMP-2 and BMP-7, induce odontogenic and osteogenic differentiation of dental stem cells. Transforming Growth Factor-β (TGF-β) regulates dentin matrix secretion and pulp repair. Fibroblast Growth Factor (FGF) helps in enhancing angiogenesis and stimulates proliferation of dental pulp stem cells. Vascular Endothelial Growth Factor (VEGF) is essential for neovascularization in pulp regeneration. Platelet-Derived Growth Factor (PDGF) promotes cell migration and proliferation, enhancing periodontal ligament regeneration. By integrating biomolecules into regenerative approaches, a more comprehensive strategy can be achieved, improving the efficacy of tissue engineering in dental applications.

## 5. Clinical Applications and Success in Regenerative Dentistry

The concept of regenerative dentistry is dynamic and holds much potential for the reconstruction of dental and periodontal tissues using technology that incorporates stem cells, biomaterials, and growth factors. Clinically, there are several potential uses of these regenerative manners with reference to tooth regeneration, periodontal regeneration, bone repair, and dental implant integration [44]. This has resulted in interest in the clinical efficacy of these treatments, showcasing certain successful cases and clinical trials of regenerative dentistry, as well as long-term prospects that influenced clinical results. The outcomes of such regenerative therapies are intrinsically related to protocols, patient selection and the incorporation of advanced technologies.

### 5.1. Clinical Studies and Success Rates in Tissue Repair

Several clinical trials have been published regarding the application of regenerative medicine in dentistry and the topic mainly deals with tissue recovering and regeneration. Clinical investigation of periodontal tissue regeneration has suggested favourable results when stem cells, growth factors including PDGF and BMPs, and scaffold matrices are employed. For instance, the properties of woody dental pulp stem cells (DPSCs) incorporated with a collagen scaffold and PDGF have been demonstrated to achieve the formation of periodontal ligament, alveolar bone, as well as cementum [45]. It is difficult to definitively report success rates for periodontal regeneration, though they are commonly reported to be between 60% and 80% for patients based on the severity of periodontal disease, age, and the quality of the material used for regeneration [46].

Several studies have revealed the potential of stem cells and biomaterials in the regeneration of alveolar bone, showing favourable changes in bone volume and density. For example, when BMSCs were mixed with bioactive ceramics, the healing and integration of bones were significantly improved in patients undergoing dental implantation [47]. The success rates for bone regeneration in clinical trials can reach up to 85% in patients with localized forms of bone defects; nonetheless, lower success rates are typical for patients with significant general health problems or extensive bone loss [48].

Tissue engineering applications for dental pulp are promising in cases of pulpitis or traumatic avulsion. Some of the clinical tried and tested techniques involve using stem cells from the apical papilla (SCAP) and bioactive scaffolds have shown clinically to partly regenerate the dental pulp with new tissue formation noted, along with enhanced pulp vitality [49]. However, replacement of functional pulp tissue remains a problem. despite the identified indications of PDLSC success in terms of neo-vascularization and tissue repair, which are still under assessment.

### 5.2. Case Reports Demonstrating Regeneration of Dental and Periodontal Tissues

Over the years, regenerative procedures have been applied in the treatment of root canal therapy in a bid to improve the status of the damaged per apical tissues among patients. Stem cells combined with scaffold material show some degree of success in regenerating the root canal space with the help of platelet-rich plasma (PRP) or growth factors. The clinical findings reveal an enhanced rate of recovery and prevention of reinfections in patients [50]. For instance, a case report described a patient showing severe periodontal attachment loss and alveolar bone and periodontal ligament absorption. The patient was treated with a regenerative approach which involved DPSCs that had been cultured on collagen hydrogels and the use of PRP for stimulation of tissues regeneration [51]. After six months of the treatment, the patient’s results demonstrated a favourable periodontal ligament regeneration, including increased levels of bone density and improved values for pocket depth. Therefore, by using DPSCs, scaffolds, and PRP collectively, we were able to achieve successful functional periodontal tissue regeneration with evidence of clinical follow-up benefits maintained.

Another case is that of a patient with severe bone resorption after tooth extraction treated with BMSCs and bioactive glass scaffolds. In the present case, the patient’s recovery period was 12 months, in which significant bone regeneration occurred, and a perfect integration of the scaffold with the new bone tissue, as seen radiographically [52]. Stem cells and bioactive materials effectively enhance the bone volume and quality of the implant site, which is beneficial for implant installation. Further, one of the case studies was that of a young patient with pulp necrosis following a traumatic injury to the tooth. In this case, the patient was treated with SCAPs, stem cells derived from the apical papilla associated with a collagen scaffold to promote pulp regeneration [53]. Examining the patient 6 months after the intervention, the evaluation showed successful revascularization, with partially restored pulp vitality and no signs of clinical infection or pulp necrosis. Radiographic imaging revealed that dentin-like tissue and increase in length of the root was successful, thus stressing the viability of tooth pulp regeneration by the application of stem cells.

### 5.3. Novel Approaches in Regenerative Dentistry

The goal of regenerative dentistry involves dental tissue restoration through the use of stem cells together with scaffolds and bioactive materials. Through stem cell technology based tissue engineering, scientists have discovered the possibility of replacing dental implants by developing root replacements. Development of optimal regenerative dentistry depends upon continued research for selecting the best stem cells and scaffold materials and cell-scaffold recombination methods. The dental follicle cell sheets (DFCSs) combined with treated dentin matrix (TDM) resulted in the development of a novel functional biological root (FBR) with a sandwich structure, which demonstrated long-term restoration of occlusal functionality during rhesus monkey testing [54]. Stem cells derived from dental follicles showed better potential for tooth cell formation and treated dentin matrix preserved its original dentinal tubules, which allowed necessary odontogenic factor release for tissue regeneration.

Stem cell-based therapies working with scaffold materials and platelet-rich plasma (PRP) enhance healing and decrease the risk of reinfections during pulp regeneration. Researchers have capitalized on dental pulp stem cells (DPSCs), bone marrow-derived stem cells (BMSCs) and stem cells from the apical papilla (SCAP) to achieve successful tissue regeneration in periodontals and pulp areas through clinical case studies, which used collagen scaffolds together with bioactive glass. Research has shown that vascularized pulp regeneration can be achieved through the use of simvastatin-functionalized GelMA cryogel microspheres containing stem cells from human exfoliated deciduous teeth [55]. The merger of nanoscale and micro-scale components leads to improved SHED cell adhesion in addition to better cell growth and odontogenic differentiation properties, endothelial cell migration and enhanced formation of vessel-rich tissue.

The regeneration of enamel proves difficult because it combines complex structural characteristics with an unrepairable nature upon tooth eruption. The available materials today do not successfully produce enamel properties that match both the mechanical behaviour and visual aspects found in natural teeth. The development of emerging strategies uses both acellular synthetic enamel reconstruction techniques alongside cell-mediated epidermal cell stimulation methods for postnatal enamel production [56]. Scientists conducting future regenerative dentistry research seek optimization strategies for clinical deployment to provide new approaches for tooth repair and replacement.

### 5.4. Challenges and Factors Affecting Clinical Outcomes

Although regenerative dentistry holds much potential, there are several clinically relevant indices that persist over the long term influencing the efficacy of these therapies. A major issue concerning dental tissue regeneration, more specifically, pulp and bone regeneration, is the capacity to attain an appropriate level of angiogenesis. It is critical for the survival of the regenerating tissues to properly form blood vessels and angiogenesis has to be efficiently induced to have proper healing and tissue viability [57]. Despite the lack of immune rejection problems when stem cells are harvested from the patient’s body, several issues can arise from foreign biomaterials or allogenic stem cells, including immune responses, inflammation or slowing of the healing process. For regenerative treatments to be effective in the long run, material–cell interaction is a paramount factor.

Additionally, the biomaterials acting as scaffolds have to degrade at an adequate pace to allow the formation of the tissue but still offer the requisite amount of mechanical support during the healing process. Scaffold design must retain control over the degradation rate; if it degrades over a short period of time, it may hinder tissue formation or, if it degrades slowly, may lead to complications, like scaffold fragmentation or failure [58].

Patient-related factors, including age and baseline health (medical illness, diabetes, smoking, autoimmune diseases, etc.) play a role in determining the chances of success in regenerative therapies. Patients in the older age group, or with comorbid conditions, may have slower healing, lesser regenerative ability, or more complications. It is critical to evaluate the tissue regeneration success not just at the end of the experiment, but also the long-term stability of the regenerated tissues [59]. This entails monitoring the functionality and integration of the regenerated periodontal ligament, bone and dental pulp for several years to ensure that the tissues have become functional and properly connected to the adjacent tissues.

Further investigation of regenerative techniques coupled with patient selection with the main focus on attention to treatment planning can help in increasing the success and predictability of the regenerative procedures used in dental practice. The advances in a number of technologies, including 3D printing, are beneficial to the evolution of direct regenerative dentistry in that they can improve the fabrication of the scaffold that surrounds stem cells and biomaterials to enhance treatment outcomes. Incorporating stem cells, biomaterials, and growth factors allows regenerative dentistry to offer more dependable, longer lasting, and customizable solutions to patients. Therefore, the number of cases and correctly designed trials will increase as the field evolves, together with the specifics of the protocols, the selection of the patient cohort, and overcoming existing hurdles.

## 6. Future Directions and Innovations

### 6.1. Emerging Technologies

The advancements that define the future of regenerative dentistry include 3D bioprinting, gene editing, and nanotechnology technologies; 3D Bioprinting technology opens the doors for creating patient-specific scaffolds and dental tissue, which can print functional tooth structures, periodontal tissues, and dental pulp. New techniques, such as CRISPR-Cas9, facilitate the improvements of stem cell regenerative capabilities, guidance of cell development, and correction of mutations [60]. Nanotechnology enables the creation of biomaterials with enhanced properties including enhanced stem cell attachment and the targeted release of drugs and growth factors for tissue repair [61]. On the same note, bioreactors are used to cultivate tissues in situ before implants, and these new bioreactors are designed to mimic real life situations in order to foster functionality of dental tissues.

### 6.2. Personalized Medicine

As applied in regenerative dentistry, personalized medicine aims at delivering healthcare interventions that are unique to the patient. This is because self-assembled stem cell therapies, where stem cells are taken directly from the affected tissue of the patient, remove the chances of rejection, since the body is unable to distinguish the difference between the newly grown cells and the pre-existing ones. Personalized regenerative treatments also include genetic tests to estimate the body’s ability for self-healing and to select the most effective remedies [62]. Innovations in technology enable the creation of structures that perfectly match the structure and size of a particular person, which in turn shortens the recovery period. Furthermore, it has been found that drug delivery systems through nano-carriers and biomaterials containing unique growth factors of patients are more efficient with fewer side effects.

### 6.3. Regulatory, Ethical Issues and Economic Implications

However, several considerations need to be made regarding the future of regenerative dentistry. Micro- and macro- regulation of stem cell therapies, gene editing, and biomaterials must respond to the speed of their evolution. These therapies have to undergo testing and trials from various regulatory authorities before they are approved to be used by everyone, which may take a lot of time and money. Ethical issues also come into the picture, for instance, concerns over the source of stem cells for use and possible consequences of genetic changes. Safe implementation can only be achieved when ethical rules are clearly spelled out and consent procedures are clear to the public. Financially, regenerative treatments are costly and this factor remains a roadblock to the implementation of the treatments, mainly in poor countries [63]. Technological developments may bring about cost reductions in the future but conscious effort must be made by researchers, clinicians, and policymakers to bring about such innovations within the populace.

## 7. Challenges

Despite the existing advancements, regenerative dentistry has several challenges and limitations that affect its implementation. Biological and technical factors might be crucial and one is the problem of stem cell availability. Certain dental tissues, like pulp and periodontal ligament, are known to harbour stem cells but sometimes may not be enough for constructing complex tissues. Vascularization is also important for tissue formation but, particularly for large graft developments, creating sufficient blood supply is a challenge [64]. Another barrier is the choice of scaffolds for tissue engineering applications; these scaffolds must be biocompatible but also mechanically strong and possess appropriate degradation profiles.

Another challenge faced is that treatments do not have standard outcomes and effectiveness because patients’ factors such as age, health condition, and genetic characteristics may have an effect. The conditions of stem cell storage and activation, cell quality, as well as an organism’s specific reactions to growth factors, create variability. There also seems to be a lack of standards for regenerative dentistry procedures. Today, it is challenging to compare the outcomes of respective studies and provide the best solution for treatment because of the lack of standards in terms of stem cell isolation, biomaterial usage, and overall treatment plan. Well-established guidelines are crucial to eliminate the variability and increase the efficacy and safely of regenerative procedures. Managing some of these challenges is pertinent to the successful implementation and deployment of regenerative dentistry in practice.

## 8. Conclusions

Stem cell and biomaterial for regeneration dentistry is one of the most promising dental technologies because of its ambitious ability to bring new and revolutionary solutions for patients. In the future, this technique will transform treatment with the aim of masking the teeth and surrounding soft tissues to a condition which aims at rejuvenating the teeth and other tissues in their functional and anatomic form; regenerative dentistry focuses on recovering damaged tissues instead of removing them, which can create lasting improvements in patient care and minimize invasiveness. Despite the current barriers in terms of biological and technical optimization techniques, the advancement made so far gives hope for exemplary dental treatments in the future. Further enhancement of these regenerative strategies holds the promise to transform the ways oral tissue repair and regeneration are implemented by dental practitioners, thus setting the stage for regenerative dentistry as a leading concept in contemporary dental practice. This proposes a transfer of the focus on the patient for the management of these cases and subsequent restoration of dental health in a manner that is biologically effective for the patient and propels the dental specialty forward.

## Figures and Tables

**Table 1:** Sources and Applications of Stem Cells in Dental Regeneration.

Stem Cells Sources	Explanation	Applications	Reference
Dental Pulp Stem Cells (DPSCs)	Derived from the dental pulp of permanent teeth.	Pulp and dentin regeneration, repair of damaged tissues in the oral cavity.	[11]
Stem Cells from Human Exfoliated Deciduous Teeth (SHED)	Harvested from baby teeth (deciduous teeth).	Formation of dentin and connective tissue, regeneration of damaged or lost dental tissues.	[19]
Periodontal Ligament Stem Cells (PDLSCs)	Isolated from the periodontal ligament of extracted teeth.	Regeneration of periodontal ligament, cementum, and alveolar bone; treatment of periodontal diseases.	[17]
Stem Cells from the Apical Papilla (SCAP)	Found in the apical region of the developing root of immature permanent teeth.	Root development, pulp and dentin regeneration, repair of immature tooth injuries.	[18]
Dental Follicle Progenitor Cells (DFPCs)	Obtained from the dental follicle of developing teeth.	Differentiation into osteoblasts and fibroblasts; regeneration of periodontal and alveolar bone tissues.	[15]

**Table 2:** Combination Strategies for Optimal Tissue Regeneration Outcomes.

Condition	Type of Stem Cell	Bioactive Material/Scaffold/PRP	Outcome	Reference
Severe Periodontal Attachment Loss	DPSCs (Dental Pulp Stem Cells)	Collagen Hydrogels + PRP	Favorable periodontal ligament regeneration, increased bone density, and improved pocket depth values	[27]
Severe Bone Resorption Post Tooth Extraction	BMSCs (Bone Marrow Stem Cells)	Bioactive Glass Scaffolds	Significant bone regeneration, scaffold integration with new bone tissue, enhanced implant site quality	[28]
Pulp Necrosis after Traumatic Injury	SCAP (Stem Cells from Apical Papilla)	Collagen Scaffold	Partial pulp vitality restoration, successful revascularization, dentin-like tissue formation, and root lengthening	[28]
Chronic Periodontitis with Bone Defects	PDLSCs (Periodontal Ligament Stem Cells)	PRP + Biodegradable Scaffold	Regeneration of periodontal ligament and alveolar bone, reduced inflammation, improved tissue attachment	[36]
Tooth Root Resorption Post-Orthodontics	SHED (Stem Cells from Human Exfoliated Deciduous Teeth)	Gelatin-Based Scaffold + VEGF	Reduced root resorption, increased cementum and periodontal tissue regeneration, improved root structure integrity	[38]

## Data Availability

The data presented in this study are available in this article.
